# Unmet need for contraception among married adolescent girls and young women in Haramaya Health and demographic surveillance system, Eastern Ethiopia

**DOI:** 10.3389/fgwh.2022.999860

**Published:** 2022-11-07

**Authors:** Saba Hailu, Nega Assefa, Tariku Dingeta, Chaltu Abdurahman, Mewardi Adem

**Affiliations:** ^1^School of Public Health, College of Health and Medical Sciences, Haramaya University, Harar, Ethiopia; ^2^Department of Public Health, College of Health and Medical Sciences, Dire Dawa University, Dire Dawa, Ethiopia

**Keywords:** unmet need, contraception, adolescents, young married women, perceived internalized stigma

## Abstract

**Background:**

The prevalence of unmet need for contraception is the highest in low- and middle-income countries (LMIC). Contraceptive use among young married or unmarried women is lower than that among older women in developing countries. Previous studies generalized the findings to all women of reproductive age and have not investigated psychosocial factors that influence contraceptive use. This study aimed to identify factors associated with unmet need for contraception among young married women in the Haramaya Health and Demographic Surveillance System (HDSS), eastern Ethiopia.

**Methodology:**

A cross-sectional, community-based study of young married women aged 15–24 years was conducted. A simple random sampling method was used to select 550 young married women. Data were collected using a pretested structured questionnaire. Using adjusted odds ratio (AOR) with a 95% confidence interval (CI), factors associated with unmet need for contraception were identified using multivariable logistic regression analysis.

**Results:**

The overall prevalence of unmet need for contraception was 154 (30.3%). Adolescents (15–19) (AOR = 2.05, 95% CI: 1.16–3.62), husbands' negative attitude toward contraception (AOR = 2.1, 95% CI: 1.05–4.46), and no previous use of contraception (AOR = 3.9, 95% CI: 2.29–6.71) were significantly and positively associated with unmet need for contraception. On the contrary, young women with secondary education or higher (AOR = 0.55, 95% CI: 0.28–1.084) were negatively and significantly associated with unmet need for contraception.

**Conclusion:**

The prevalence of unmet need for contraception among young women in Haramaya was high. Unmet need was affected by age, husbands' attitude toward contraceptives, the educational status of women, and previous use of contraception. This study underscored the need to improve girls' educational status to empower them in making contraceptive use decisions with their partners. Programs should also engage male partners who are perceived as key decision-makers when it comes to contraceptive use.

## Background

There are more young people in the world today than ever before; nearly one in every five people around the world are between the ages of 15 and 24 (1). The World Health Organization (WHO) defines “adolescents” as individuals aged 10−19, and “young people” as people aged 10–24 (2). The majority of young people live in developing countries, and the top 10 countries with the youngest populations are all in sub-Saharan Africa (SSA). The young women in poor settings face a variety of sexual and reproductive health (SRH) problems, such as unintended pregnancies, high fertility, and abortion (3).

Family planning (FP) has been considered an essential factor in fertility regulation and in the reproductive health of women, particularly among adolescent girls and young women who want to space or limit childbearing (4). However, African countries still experience relatively high fertility rates due to women's early sexual debuts as a result of early marriage and unmet need for FP (5). Unmet need for contraception is the gap between women's desire for FP and their use of contraception methods (6). According to the WHO, women who indicate that they do not want another child or would like to postpone the next birth for at least 2 years but are not using any method of contraceptive are referred to have an unmet need for FP (7).

Contraceptive use among young married and unmarried women (aged 15–24) is lower than that among older women in the developing world. The prevalence of unmet need is the highest in the West and Central African regions (29.3% among young married women and 41.7% among young unmarried women), and a high prevalence of unmet need corresponds to the heightened risk of unwanted pregnancy and related morbidities and mortalities (1, 8).

Serious pregnancy and childbirth complications can lead to maternal death among adolescent girls and young women. Globally, it is estimated that 15% of maternal deaths occur among young women aged 15–25. Teen mothers are at high risk of adverse pregnancy outcomes like obstructed labor, low birth weight, and perinatal death (9, 10). Youth mortality can be related to a high burden of unintended pregnancy, most likely due to limited access to FP services, and the risk is much higher when pregnancy is unintended. Nearly half (45%) of the pregnancies among adolescents in developing countries are unintended, and more than half result in abortion, often under unsafe conditions and miscarriages (11). Approximately 23 million people have an unmet need for modern contraception, increasing their risk of unintended pregnancy. Meeting the need for modern contraception among adolescents in low- and middle-income countries (LMICs) would avert 2 million unplanned births, 3.2 million abortions, closely spaced births, 5,600 maternal deaths among adolescent girls, and infant mortality each year (11).

Despite efforts to revitalize FP programs, it is imperative to address the growing need for contraceptives among African youth in particular. Data on the need for pregnancy spacing indicate that FP needs of youth are 2.3 times higher than those of the adult population (1). The FP 2020 initiative strongly committed to prioritize the needs of young women in their FP and SRH programs (12). Currently, Ethiopia is committed to increasing contraceptive use among 1 million adolescents and youth by 2025 through the smart start program. The program uses financial planning as an entry point to engage young married couples in planning their futures and achieving financial stability, positioning contraception as a tool to help them achieve their self-defined goals (13).

Previous research conducted among reproductive-aged women in Ethiopia showed that individual factors such as socioeconomic status, age, parity, and spousal communication, as well as household decision-making and discussion with healthcare providers affect FP use (14–17). There are several gaps in the literature on unmet need among young women in the developing world. Despite extensive research, studies neglected and hardly mentioned the importance of examining psychosocial factors in contraceptive use. Sociocultural and structural barriers often prevent young women from achieving their reproductive intentions (18). Improving availability, affordability, and youth-friendliness may not fully address the psychosocial barriers to contraceptive use among them (19). Additionally, researchers generalize contraceptive use among all women; however, recent studies emphasized the need of age differentiation when studying factors influencing contraception use. Due to the different fertility preferences associated with this stage of the life course, young women may have a disproportionately unmet need for FP. Hence, the objective of this study is to assess factors influencing unmet need for contraception among young married women in the Haramaya Health and Demographic Surveillance System (HDSS), eastern Ethiopia.

## Methods

### Study design and setting

From February to March 2020, a community-based cross-sectional study was conducted in Haramaya HDSS, which is located in Haramaya district (administrative divisions), East Hararghe Zone, Oromia Regional State. Haramaya district is located 500 km away from Addis Ababa, the capital city of Ethiopia. Haramaya district has 36 rural kebeles (the smallest administrative unit in Ethiopia) and five urban kebeles, as well as eight health centers and one general hospital. The Haramaya health and demographic health survey (HDSS) field site, maintained by Haramaya University, is located in the 12 rural kebeles of Haramaya district. The site has 93,363 residents and 12,829 married reproductive-age women. Of these, 18% were young women aged 15–24.

### Population and sampling technique

The study population included all young married women aged 15–24 who lived in the study area. The sample size was calculated using the single population sample calculation formula, and the prevalence of unmet need for contraception among married young women from a previous study in eastern Ethiopia was 34.6% (20). The sample size for the associated factors of unmet need for contraception was also determined using two population proportion formulas in EPI INFO version 7 software, with the assumption of a two-sided confidence level of 95%, a 5% margin of error, a power of 80%, and the ratio of exposed to unexposed of 1:1. The maximum sample size of 500 was reached during the double population formula, and a 10% non-response rate resulted in a final sample size of 550.

A multi-stage sampling technique was used, with kebeles as primary sampling units (PSUs) and households as secondary sampling units (SSUs). The study included 12 kebeles, two of which were selected using the lottery method. After obtaining the sampling frame from the Haramaya HDSS database, the sample size was proportionally distributed to the selected kebeles. Finally, a simple random sampling technique was used to select households in the kebele to be visited for data collection. Finally, young married women aged 15 to 24 were recruited from the selected households.

### Data collection and variable specification

Data were collected by trained data collectors using a semi-structured questionnaire. The tool was adapted from the related literature and based on filtered questions from the Ethiopian Demographic Health Survey (EDHS) 2016 with some modifications. The tool and the questionnaire were prepared in English, translated into a local language (Afan Oromo), and translated back to English by a linguist for consistency. The questionnaire was pretested on 5% (28) of participants in Kersa HDSS before the initiation of the study. Data were collected by six women data collectors and two field supervisors. Before the initiation of the study, a 2-day training on the contents of the questionnaire, data collection techniques, and research ethics was provided. Daily checks were conducted by the assigned supervisors for completeness, consistency, and accuracy of the data in the data collection period.

### Data analysis methods

Prior to data entry, all questionnaires were checked for completeness by a principal investigator and supervisors. It was coded and entered into Epi-Data version 3.1 before being exported to the STATA version 16 statistical package for analysis. For categorical variables, a univariate analysis (frequencies and percentage) was performed, and means were calculated for continuous variables. Narration, tables, and graphs was used to present the results. Binary logistic regression analysis was used to identify factors associated with the outcome variable. Multivariable binary logistic regression analyses were performed on those variables used in bivariate analysis with a *p* < 0.25. To measure the strength of the association between the outcome and independent variables, the odds ratio with a 95% confidence interval (CI) was computed. Finally, in binary logistic regression, variables with a *p* < 0.05 were considered statistically significant. Hosmer–Lemeshow goodness-of-fit test was used to evaluate the model fitness. The multi co-linearity test was performed to observe the correlation between independent variables.

### Outcome variables and measurements

**Figure 1:**
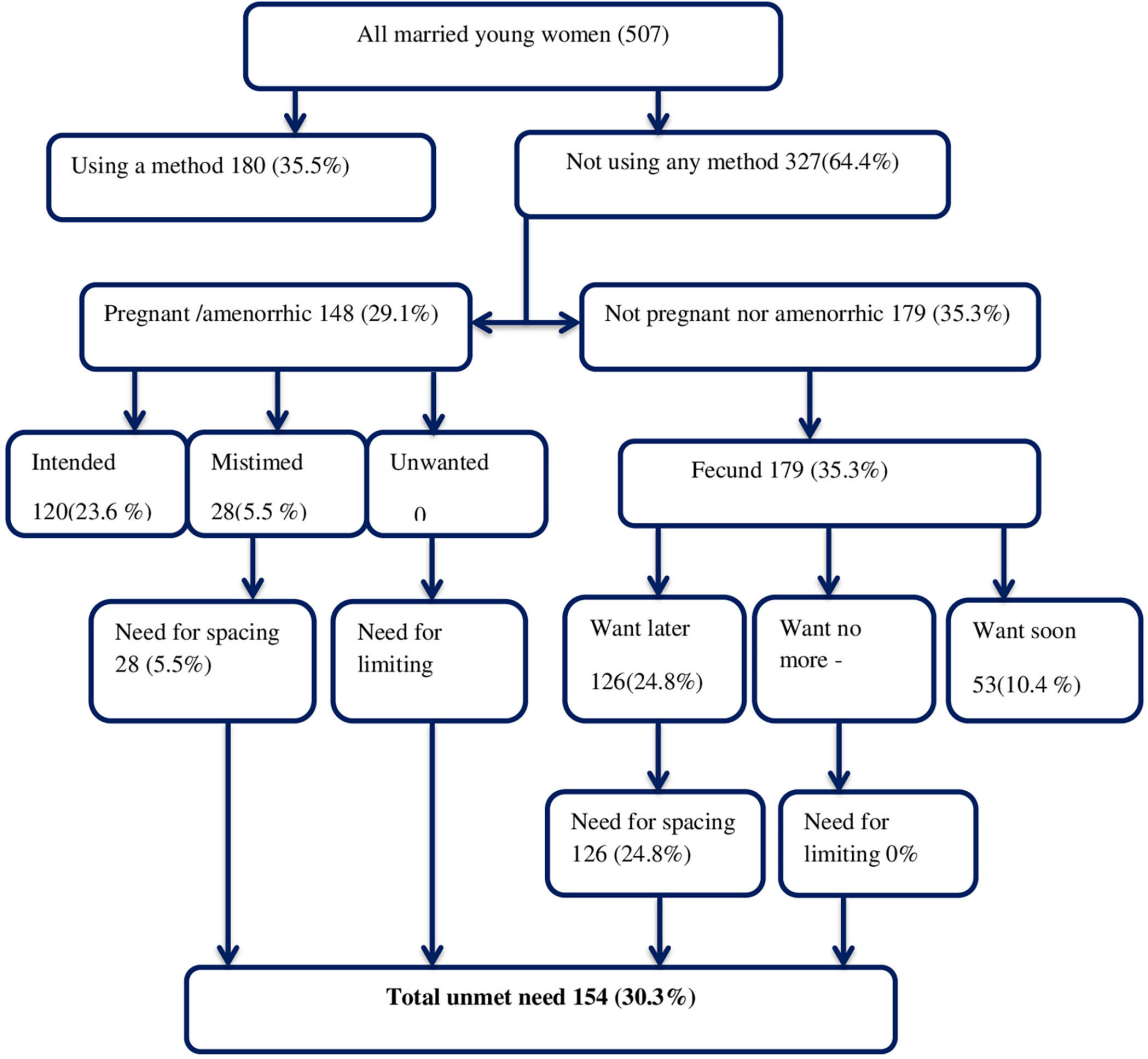
Algorithm of unmet need for contraception among young married women in the Haramaya HDSS, eastern Ethiopia, 2020.

The main outcome variable in this study is the unmet need for contraception, which is computed using the following steps (Figure 1).

**Step 1:** Contraceptive use status—the percentage of young married women who did not use contraception was determined.
**Step 2:** Pregnancy and amenorrhea status—women in step 1 were divided into two groups (pregnant or amenorrhea and non-pregnant or amenorrhea).
**Step 3:** Based on the desire for pregnancy status from the two identified groups in step 2, three other percentages were developed (pregnancy mistimed, pregnancy unwanted, and intended pregnancy).
**Step 4:** Future fertility intentions for the fecund group were considered, and the percentages of those who want to postpone childbearing (want later/spacers), those who are unsure if or when they want, and those who want to limit (want no more children/limiters) were computed.
**Step 5:** The percentage of women who have an unmet need for spacing and limiting was calculated for the groups identified in steps 3 (pregnancy mistimed and pregnancy wanted) and 4 (the proportion of fecund women who want childbearing later, those who are unsure if or when they want, and those who want no more). The sum of proportions of unmet need for spacing and limiting gave the final unmet need for contraception.

The family wealth index was calculated using the principal component analysis (PCA) method. Items were assessed based on household assets and the number of animals owned, and each household was assigned to one of the five categories (lowest, second, middle, fourth, and highest).

Perceived internalized stigma toward FP use, an independent variable identified as a predictor of unmet need for contraception among young married women, is an index to be measured using the combination of five questions related to fear, worry, and embarrassment when accessing FP from the literature (21). The five items include “I would feel embarrassed about wanting more information about FP service,” “I would be afraid of being seen by someone I knew at the facility,” “I would be worried about what my parents would say if they found out that I needed FP services,” “I would be worried about what people in my community would say about me if they found out I needed FP,” and “I would feel embarrassed talking to a provider about FP.”

The responses were classified as strongly disagree, disagree, neutral, agree, and strongly agree. The total score was then obtained by summing all items with values ranging from 5 to 25 points and categorizing the result as above and below the median. Those with scores above the median were classified as having perceived internalized stigma for contraceptive use, while those with scores below the median were classified as not having perceived internalized stigma for contraceptive use.

The five items in the index were combined to form a composite score ranging from 1 to 5, with a higher score indicating a higher level of perceived stigma. With Cronbach's α = 0.96, the scale had good reliability.

### Operational definitions

*Women with unmet need*: The proportion of women who (1) are not pregnant, do not have postpartum amenorrhea, and want to postpone their next birth for 2 or more years (unmet need for spacing) or stop childbearing altogether (unmet need for limiting) but are not using a contraceptive method, or (2) have a mistimed or unwanted current pregnancy, or (3) have postpartum amenorrhea and have had their last mistimed or unwanted birth in the last 2 years (unmet need for contraception) (22).

Another explanatory variable was contraceptive knowledge, which was assessed through 12 questions adapted from the EDHS 2016 related to types of contraceptives. Response for each question was coded as 1 for “Yes” answers and 0 for “No” answers. The score was computed by summing all of the items ranging from 0 to 12 for each participant; then, a composite knowledge variable was computed from the score using the median as a cutoff point. Participants with mean or higher scores were classified to have “good knowledge,” while those with scores below the mean were categorized to have “poor knowledge.” The internal consistency (α) of the items was checked and found to be 0.74, indicating an acceptable level of reliability.

## Results

### Sociodemographic characteristics

Of 550 sampled participants, 507 responded to interviews and gave complete data, for a response rate of 92.1%. The mean age of the respondents was 20.9 ± 2.4 years. The majority of the respondents, 371 (73.2%), were 20–24 years of age, and 486 of them (95.8%) were Muslim. Regarding the educational status of respondents, 224 (44.2%) and 193 (38.1%) of their husbands had no formal education. More than two-thirds of the participants were housewives, and one-third of the participants were in the poor wealth quintile (Table 1).

**Table 1:** Sociodemographic characteristics of young married women in the Haramaya HDSS, eastern Ethiopia, 2020.

**Characteristics**	**Categories**	**Frequency**	**Percentage (%)**
Age	15–19	136	26.8
	20–24	371	73.2
Religion	Muslim	486	95.8
	Others^*^	21	4.2
Educational status of women	No formal education	224	44.2
	Primary school education	187	36.9
	Secondary school education and above	96	18.9
Women occupation	Housewife	366	72.2
	Merchant	67	13.2
	Student	28	5.5
	Government employee	35	6.9
	Farmer	11	2.2
Husband's educational status	No formal education	193	38.1
	Primary school education	169	33.3
	Secondary school education and above	145	28.6
Husband's occupation	Farmer	358	70.6
	Merchant	77	15.2
	Government employee	51	10.1
	Other^*^	21	4.1
Wealth quintile	Lowest	102	20.1
	Second	101	19.9
	Middle	102	20.1
	Fourth	101	19.9
	Highest	101	19.9

* “Other” husband occupation category includes daily laborer and no job, and religion category includes orthodox and protestant.

### Reproductive history

The age of the respondents at the first marriage ranges from 13 to 24 years. Women married at the mean age of 17.5 ± 2.1. Of 507 young married women, 439 (86.5%) women have ever been pregnant. The mean age of the respondents at the time of first delivery was 18.3 ± 1.9 years. Of the 507 young married women, 31 (7.1%) and 35 (7.9%) of the respondents had a history of child loss and abortion, respectively. The mean desired number of children among the respondents was 6.2 children, 301 (60.2%) young women sought more than six children in their lifetime, and seven (1.4%) women had not decided yet. During the study period, 89 (17.5%) were pregnant (Table 2).

**Table 2:** Reproductive characteristics of young married women in the Haramaya HDSS, eastern Ethiopia, 2020.

**Variables**	**Categories**	**Frequency**	**Percentage**
Age at marriage	< 18	241	47.5
	≥18	266	52.4
Number of pregnancy	0 (never been pregnant)	68	13.4
	Pregnant one time	172	33.9
	Two and above	267	52.3
Number of live birth	0 (No child)	99	19.5
	One birth	178	35.1
	Two and above	230	45.3
History of dead child (*n* = 439)	Yes	31	7.1
	No	408	92.9
History of abortion (*n* = 439)	Yes	35	7.9
	No	404	92.1
Desired number of children	0	–	–
	1–3	37	7.3
	4–5	162	31.9
	≥6	301	59.3
	Did not decide	7	1.3
Pregnant/postpartum amenorrhea	Yes	148	29.1
	No	359	70.8
Current pregnancy/last birth (*n* = 148)	Intended	120	23.7
	Mistimed	28	5.5
	Unwanted	–	

### Knowledge and use of contraceptives

Approximately 85% of study participants were familiar with at least one method of contraception (Table 3). The most commonly known contraceptive methods were injectable used by 356 (82.79%) participants, implant by 325 (75.5%), and pills by 313 (72.7%), while only 29 (6.7%) knew emergency contraception. Meanwhile, natural methods, such as the calendar method, and withdrawal were mentioned by 33 (7.6%) and 8 (1.8%) of young married women in the study area, respectively. A total of 292 (57.6%) respondents have poor knowledge of contraceptives, and 215 (42.4%) have good knowledge of contraceptives. However, 241 (47.5%) have ever used one or another type of contraceptives. The most favorite modern contraceptive ever practiced by young married women was injectable, used by 139 of the participants (57.6%).

**Table 3:** Knowledge and general information of contraception among young married women in the Haramaya HDSS, eastern Ethiopia, 2020.

**Variables**	**Categories**	**Frequency**	**Percentage**
Ever heard about contraceptives	Yes	430	84.8
	No	77	15.2
Do you know a place where you can get contraceptives (*n* = 430)	Yes	396	92.1
	No	34	7.9
Discussed family planning with health providers in the past 12 months (*n* = 430)	Yes	275	67.9
	No	155	32.1
Discussed about contraception with husband (*n* = 430)	Yes	256	50.4
	No	251	49.5
Perceived Husbands' attitude (*n* = 256)	Supportive	179	69.9
	Not supportive	75	29.2
	I do not know	2	0.8
If not supportive what's his reason (*n* = 75)	Religious believes	48	64.8
	Wants more children	17	22.9
	Not aware of contraceptive	4	5
	I do not know	6	8.1

### The magnitude of the unmet need for contraception

Of 359 women, 127 (25.1%) did not need contraception because they planned to have a child soon. Contraceptive needs of 180 (35.5%) women were met because they used FP methods. The prevalence of unmet need among young married women in this study was 154 (30.3%, 95% CI: 26.3–34.4), with all having an unmet need for spacing (Figure 1).

### Perceived internalized stigma

**Figure 2:**
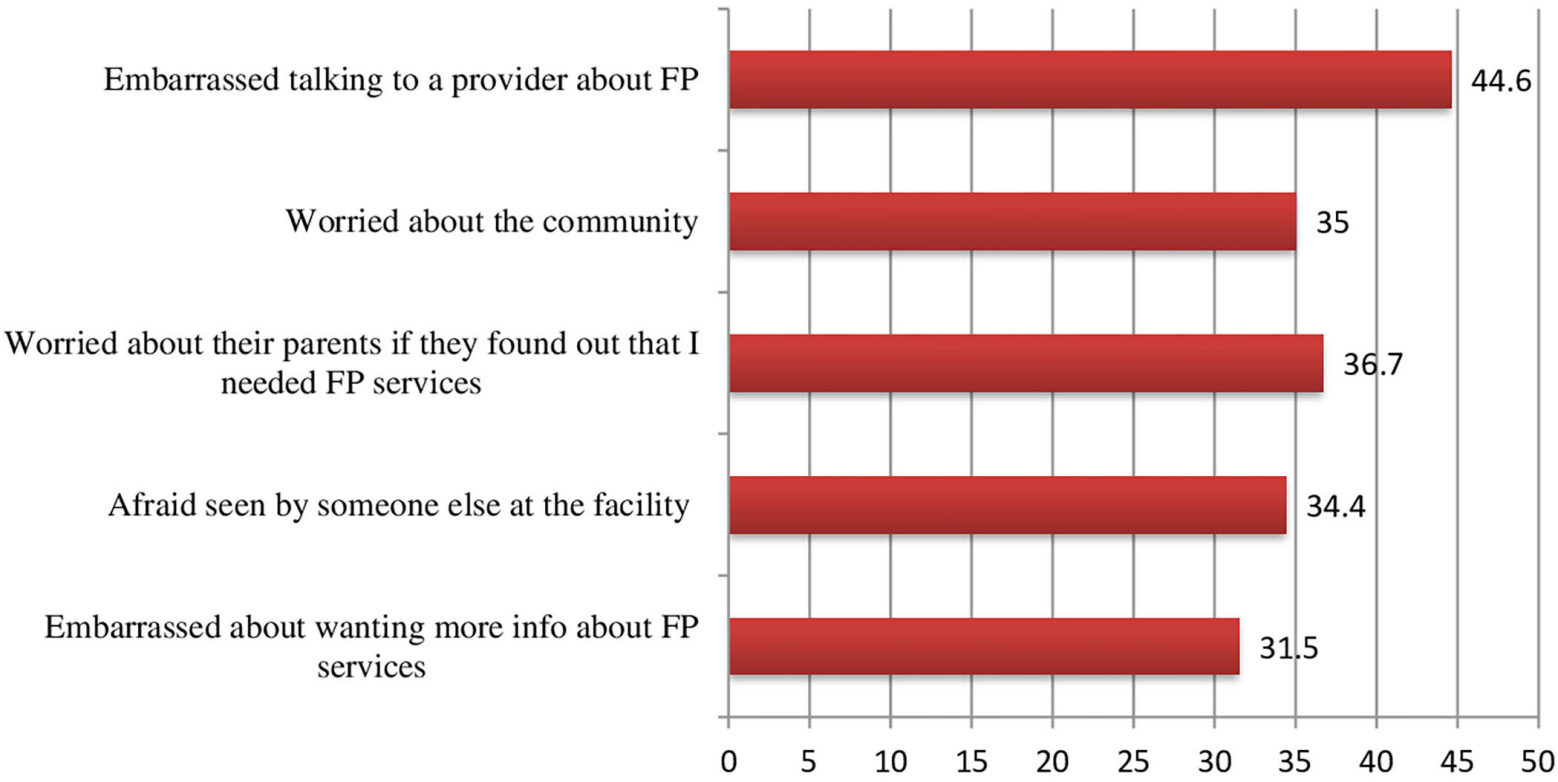
Agreement with perceived internalized stigma statements by 15–24-year-old young married women.

The percentage of young women who agreed with five of the stigma questions is illustrated in Figure 2. Approximately 31% of young women agreed that wanting more information about FP services would embarrass them. Approximately 34% agreed that they were concerned about being seen by someone else at the facility, and 36.6% of them were concerned about their parents finding out that they needed FP services. Approximately 35% of young women are concerned about the community, and 44% of young women are embarrassed to discuss FP with a provider (Figure 2).

### Factors associated with unmet need for contraception

In the analysis of bivariable logistic regression, variables with a *p* < 0.25 were women's age, respondent's educational status, women's occupation, discussion about FP with a health provider in the past 12 months, husbands' attitude toward contraception, the knowledge of contraception, ever use of contraceptives, age at the first marriage, the number of living children, and perceived internalized stigma to FP use (Table 4).

**Table 4:** Bivariate and multivariate binary logistic regression analyses for unmet need for contraception and associated factors among young married women in the Haramaya HDSS, eastern Ethiopia, 2020.

**Variables**	**Unmet need**		**Crude OR**	**Adjusted OR**
	**Yes**	**No**		
**Age of women**				
15–19	59 (43.3%)	77 (56.6%)	2.22 (1.47–3.35)	2.05 (1.16–3.62)
20–24	95 (25.6%)	276 (74.3%)	1	1
**Women educational status**				
No formal education	81 (36.1%)	143 (63.9%)	1	1
Primary school education	54 (28.9%)	133 (71.1%)	0.7 (0.47–1.08)	0.64 (0.37–1.11)
Secondary school education and above	19 (19.8%)	77 (80.2%)	0.43 (0.24–0.77)	0.42 (0.19–0.93)
**Wealth quintile**				
Lowest	28 (27.4%)	74 (72.6%)	1	1
Second	34 (33.6%)	67 (66.4%)	1.34 (0.73–2.44)	1.03 (0.46–2.29)
Middle	31 (30.3%)	71 (69.7%)	1.15 (0.62–2.11)	0.73 (0.32–1.68)
Fourth	29 (28.7%)	72 (71.3%)	1.06 (0.57–1.96)	0.64 (0.28–1.44)
Highest	32 (31.6%)	69 (68.4%)	1.22 (0.66–2.24)	0.65 (0.29–1.47)
**Knowledge on contraception**				
Poor knowledge	92 (31.5%)	200 (68.4%)	1	1
Good knowledge	62 (28.8%)	153 (71.2%)	0.8 (0.81–0.96)	1.08 (0.62–1.90)
**Age at marriage**				
<18 years	83 (34.3%)	159 (65.7%)	1	1
≥18 years	72 (27.1%)	194 (72.9%)	0.7 (0.49–1.03)	1.2 (0.71–2.22)
**Number of live birth**				
Never gave birth	32 (32.3%)	67 (67 6%)	1	1
One birth	57 (32.0%)	121 (67.9%)	0.52 (0.29–0.94)	0.81 (0.42–1.56)
Two and above birth	65 (28.4%)	164 (71.6%)	0.48 (0.27–0.83)	1.05 (0.52–2.13)
**Ever used contraception**				
Yes	31 (12.8%)	210 (87.1%)	1	1
No	123 (46.3%)	143 (53.7%)	5.8 (3.72–9.11)	3.85 (2.24–6.59)
**Husbands attitude toward contraception**				
Supportive	24 (13.4%)	155 (86.6%)	1	1
Not supportive	25 (33.4%)	52 (66.6%)	3.1 (1.63–5.90)	2.35 (1.12–4.92)
**Perceived internalized stigma toward FP use**				
Yes	45 (26.9%)	122 (73.1%)	0.6 (0.45–1.01)	0.71 (0.45–1.14)
No	109 (32.0)	231 (67.9%)	1	1

Variables that were significant in the bivariate analysis were selected and included in the multivariable analysis to control for possible confounders on unmet need for contraception. In the multivariable analysis, the age of women, women's educational status, husbands' attitude toward contraception, and ever use of contraception were all significantly associated with unmet need for contraception. Married adolescents (15–19 years) were 2.05 times more likely to have an unmet need for contraception than married women in the 20–24 age groups [adjusted odds ratio (AOR) = 2.05, 95% CI: 1.16–3.62]. Women with secondary and higher education (AOR = 0.55, 95% CI: 0.28–1.084) were 45% less likely to have an unmet need for contraception than women with no formal education. Husbands' attitude is also significantly associated with unmet need for contraception. Women whose husbands have no supportive attitude toward contraception were 2.1 times more likely to have an unmet need for contraception than their counterparts (AOR = 2.1, 95% CI: 1.05–4.46). Women who had never used contraceptives before were 3.9 times more likely to have an unmet need than women who had ever used contraceptives (AOR = 3.9, 95% CI: 2.29–6.71).

## Discussion

Regional estimates from SSA showed comparable results, with the prevalence of unmet need ranging from 14.7 to 45.7% among young married women, for example, unmet need among young married women in Eritrea (34.8%), Kenya (30.2%), and Uganda (34.3%) (23). This is due to the SRH services failing to meet the needs of the sub-Saharan young population (24). The prevalence of this study was consistent with prior research conducted in rural Tigray: where it was 32.5% among married adolescents and 28% among youth aged 20–24; in Jigjiga city, where it was 29%, and in Kersa, where it was 34.5% among young married women (25). This might be due to the sociocultural similarities between the study areas. Even when young women desire and are willing to use modern contraception methods, such services are sometimes severely hindered by their partners' preferences or negative attitude toward contraceptive use, as evidenced in our study (1). Lack of support from social or norms groups could be a possible explanation for not using contraception; thus, studies showed that social support is the most important motivator of contraceptive use (26). A positive attitude from peers and approval from friends and families encourage young people to use contraception (27).

Unmet need for contraception is significantly higher among married adolescents (15–19) than among young women aged 20–24. This may be due to the immaturity of married adolescents (15–19 years of age), particularly regarding lack of knowledge on contraceptives. As evidenced by the study, more than half of young women (57.6%) have limited knowledge of contraceptive methods. Several studies found that adolescents tend to have lower contraception use and knowledge, as well as less access to information and services than older women (28, 29). Knowledge and awareness can help adolescent decision-making by expelling misconceptions and clarifying questions. Yet, adolescents tend to be poorly informed about reproductive matters (30). Another explanation could be that belief in erroneously perceived side effects and myths shred by significant others might severely hinder contraceptive utilization among adolescent girls (27, 31, 32). In the meantime, in rural Ethiopia, young women get married despite having limited information of FP (33). In our study, 47.5% of the study participants married before the age of 18. Several studies highlighted that, even if adolescents want to use a contraception method, social pressure, particularly from in-laws, may prevent them from using contraceptives. This is because young women are under pressure to conceive and bear children soon after marriage to prove their fertility, begin adulthood, secure their marriage, and gain respect (34, 35).

In the current study, a statistical association was established between husbands' attitudes and unmet needs. A woman whose husband had a non-supportive attitude toward contraception was 2 times more likely to have an unmet need than a woman whose husband had a supportive attitude. Our results are consistent with the results of previous studies in the Awi zone, Ethiopia, and Cameron: partner approval and support was the main determining factor in girls' FP use (15, 36). This is possible because husbands are perceived as the key decision-makers when it comes to contraception. For younger women, the perception that their husband/partner supports FP had a positive impact on their contraceptive use (19). Other plausible reasons might include gender inequitable norms affecting contraception use among young married women. Young women whose husbands do not support contraceptive use have limited participation and control in decision-making in a marital relationship and low negotiation (37). Conversations with intimate partners often resulted in consent for contraceptive use (38). We did find that 51% of young wives in our sample reported that they did not have a spousal discussion about contraception with their partner. Given that husbands were influential in contraceptive utilization, engaging men in an intervention that averted gender inequalities in making contraceptive use decisions by providing culturally suitable information on contraception, and encouraging spousal communication might be effective.

This study found that girls' educational status is associated with unmet need for contraception, indicating that young women with secondary and higher educational levels have lower odds of having an unmet need for contraception than young women with no formal educational status. The association between educational level and unmet need has been reported in other studies. According to the EDHS, in Ethiopia and Swaziland, unmet need declines with educational attainment for young women aged 15–24 (39). This association was also consistent with previous studies conducted in Sudan (40) and Malawi (41). The plausible reasons may be related to the educational status of women, which influences fertility and imparts skills that can alter how women perceive their role in society. An increase in education may be related to being aware of reproductive rights and power in making a contraceptive decision with their spouse, which would reduce the burden of unmet need for contraception. Education for women improves the reproductive life of women by enhancing and empowering them within the family and in their relationship with men (42). Additionally, education for young women and increased contraceptive use among adolescents and young women are two targets for the sustainable development goals; therefore, the observed association is related to the current program (43). However, an inverse relationship was observed in a study conducted among SSA countries (4) and Nepal (44). Since SRH education is not provided among rural schools, young women may not be exposed to adequate SRH information during their school years. Further investigation should be implemented to explain the discrepancy.

Previous use of FP by women was also found to be significantly associated with unmet need for contraception. The odds of having an unmet need for contraception among currently married women who had never used contraception before was three times more likely compared to married women who had ever used FP before. Research conducted in Shire Endaselassie, North Ethiopia, and Tiro Afeta District, South West Ethiopia (25, 45) support this. This is possible because previous users are accustomed to the service and receive information from the health providers. This enables them to have adequate FP knowledge, leading to an increment in the practice of contraceptive methods.

Even though the association between perceived internalized stigma and unmet need for contraception has been reported in other studies, it is lost in multivariate analysis in this study. The inconsistent findings may be due to socio-cultural differences. Studies incorporating qualitative methods are essential to explore the experiences of adolescents and young women regarding contraceptive use.

## Limitations

Only married young women were included in this study; therefore, the result of this study might not be representative of the whole youth population. The study might be subject to recall bias, which might underestimate the burden of unmet need for contraception in this population. Because the pregnancy status of young women was collected through self-report, the prevalence of unmet need for contraception was overestimated and underestimated. Additionally, due to the sensitive nature of some questions, this study may not rule out the social desirability bias.

## Conclusions

This study underscored the need to improve girls' educational status to empower them in making contraceptive use decisions with their partners. Programs should also engage male partners because they are perceived as key decision makers when it comes to contraceptive use. Because most women in this study were subjected to early marriage, the local government should make efforts to create awareness in rural areas and implement the legal marriage age.

## Data availability statement

The original contributions presented in the study are included in the article/supplementary material, further inquiries can be directed to the corresponding author.

## Ethics statement

The study was conducted following the declaration of Helsinki where ethical approval was obtained from the Haramaya University College of Health and Medical Sciences Institutional Health Research Ethics Review Committee (IHRERC). For respondents aged <18 years oral assent was also obtained from the parent or guardian of the respondents. Informed, voluntary, written, and signed consent was obtained from each study participants.

## Author contributions

SH carried out the overall design and execution of the study, as well as statistical analysis, and writing of the manuscript. NA and TD critically revised the design of the study, data collection techniques, and helped with the statistical techniques. CA and MA worked on the statistical analysis and manuscript development. All authors read and finally approved this manuscript before it was submitted.

## Funding

SH received funds for data collection from Haramaya University.

## Conflict of interest

The authors declare that the research was conducted in the absence of any commercial or financial relationships that could be construed as a potential conflict of interest.

## Publisher's note

All claims expressed in this article are solely those of the authors and do not necessarily represent those of their affiliated organizations, or those of the publisher, the editors and the reviewers. Any product that may be evaluated in this article, or claim that may be made by its manufacturer, is not guaranteed or endorsed by the publisher.

## Abbreviations

AOR: adjusted odds ratio
CI: confidence interval
COR: crude odd ratio
CPR: contraceptive prevalence rate
EDHS: Ethiopian Demographic Health Survey
FMOH: Federal Ministry of Health
FP: family planning
HDSS: Health Demographic Surveillance System
IHRERC: Institutional Health Research Ethics Review Committee
LMICs: low- and middle-income countries
SRH: sexual and reproductive health
SSA: Sub-Saharan Africa
WHO: World Health Organization.
